# Exploring the impact on primary care mental health practitioners of the death of a patient by suicide: An IPA study

**DOI:** 10.1111/papt.12426

**Published:** 2022-09-19

**Authors:** David M. Sandford, Olivia J. Kirtley, Richard Thwaites, Rory C. O'Connor

**Affiliations:** ^1^ University of Central Lancashire Preston UK; ^2^ Suicidal Behaviour Research Laboratory University of Glasgow Glasgow UK; ^3^ Center for Contextual Psychiatry KU Leuven Leuven Belgium; ^4^ First Step, Cumbria, Northumberland, Tyne and Wear NHS Foundation Trust Cumbria UK

**Keywords:** impact, Improving Access to Psychological Therapies (IAPT), IPA, suicide

## Abstract

**Objectives:**

There have been numerous qualitative studies into the impact of the death of a patient by suicide on clinicians, but the majority of studies have focussed on psychiatrists and psychologists, primarily in inpatient or secondary care settings. To date, little has been done to explore the impact of such deaths on other mental health practitioners working in primary care, such as those working in Improving Access to Psychological Therapies (IAPT) services.

**Design:**

This qualitative study used purposive sampling and adopted an interpretative phenomenological analysis (IPA) methodology.

**Method:**

All participants had experienced the death of a patient in their role as a practitioner in an IAPT service. Seven practitioners were recruited from services across the North of England. Semi‐structured, one‐hour telephone interviews were audio recorded and then transcribed verbatim.

**Results:**

Analysis of the transcripts identified a number of themes, which were represented in the majority of cases. Specifically, the analyses yielded four superordinate themes: (1) feeling shocked and upset about the death of a patient; (2) attempting to understand the causes of the suicide; (3) learning from the tragic event; and (4) reflections on what helped in coping with the tragic event. The emotional responses of shock, upset, guilt and fear of blame by IAPT practitioners following the death of a patient through suicide is consistent with that found in studies of other mental health practitioners.

**Conclusions:**

It is hoped that the current study will help raise awareness amongst primary care mental health practitioners, services and training centres, of the impact of losing a patient to suicide and will encourage them to consider how best to prepare and support practitioners in this eventuality. Recommendations include raising awareness of the potential for patient suicide in primary care services, providing clarity on the individualised support available and on the requirements of investigations.

**Practitioner points**

- Be aware of the likelihood of experiencing a patient suicide. (i.e. the ‘career prevalence’ of such an event).
- Have regular training in risk assessment, formulation and safety planning, including recognition of the poor predictive power of risk assessment tools and the importance of the therapeutic encounter to enable open discussion of risk.
- Receive clear guidance on self‐care and support available in the event of a patient death and have an understanding of the service and statutory procedures following a suicide.
- Benefit from a clear procedure for communicating to the practitioner in the event of a patient death (by those with direct management or clinical responsibility for that person) in a caring and supportive manner.

## INTRODUCTION

It is estimated that 135 people are impacted to some degree by each suicide death (Cerel et al., 2016). Approximately one third of people who die by suicide have been in contact with mental health services in the preceding 12 months (National Confidential Inquiry into Suicide and & Homicide by People with Mental Illness, 2014) and clearly, mental health practitioners working in these services will be amongst those affected when an individual dies by suicide.

Problems with access to appropriate mental health care are recognised worldwide (World Health Organization, 2019). There have been many attempts to address this, including the integration of mental health services within general health care settings, that target populations affected by adversity and the dissemination of scalable psychological interventions (Ghebreyesus, 2019). The English Improving Access to Psychological therapies (IAPT; Clark, 2011) services are part of an initiative to increase access to mental health care, which has informed programmes in New Zealand (Haarhoff & Williams, 2017), Norway (Knapstad et al., 2018), Australia (Baigent et al., 2020), Canada (Naeem et al., 2017) and Japan (Kobori et al., 2014). IAPT services are characterised by a stepped care structure to deliver evidence‐based interventions at scale (Wakefield et al., 2021). This has seen the development of a new mental health workforce to support people experiencing common mental health problems such as depression or anxiety disorders. The workforce comprises of Psychological Wellbeing Practitioners (PWPs), trained to deliver guided self‐help based on cognitive behavioural therapy (CBT) approaches and High Intensity practitioners who are largely trained CBT therapists.

Individuals identified as being at significant risk of suicide would require a wider and more intensive package of care such as that provided by a multi‐disciplinary team (National Institute for Health and Care Excellence, 2011). However, IAPT staff will have contact with people at risk of suicide, given that access to IAPT services can be by self‐referral, risk is dynamic and can change during the course of treatment, and risk may only be identified once a therapeutic relationship has been established. Although there are no national data on the numbers of suicides of patients in contact with IAPT services, the sad reality is that patient suicides occur. These result in internal investigations and inquest procedures, which have an impact on staff.

There have been numerous investigations into the impact of losing a patient to suicide on mental health practitioners, but the majority have focussed on psychiatrists and psychologists (Alexander et al., 2000; Foley & Kelly, 2007; Lafayette & Stern, 2004; Sandford et al., 2020), primarily in inpatient or secondary care settings. Studies have often reported on both the personal and professional impact (Lafayette & Stern, 2004). Quantitative studies have employed the Impact of Events Scale (IES or IES‐R, Horowitz et al., 1979; Weiss & Marmar, 1996) and reported clinically significant scores (Chemtob et al., 1988; Ruskin, 2004; Yousaf et al., 2002). Common responses to losing a patient to suicide described in qualitative studies include stress, guilt, symptoms of post‐traumatic stress disorder, fear of litigation and retribution, and a more defensive approach to managing risk (Foley & Kelly, 2007). The reaction has been characterised as one of prolonged grief (Darden & Rutter, 2011), with a sense of failure and self‐scrutiny also having been highlighted (Davidsen, 2011). Qualitative studies have explored the impact on psychiatrists (Cotton et al., 1983), psychiatry trainees (Dewar et al., 2000), psychologists (Darden & Rutter, 2011), psychotherapists (Goldstein & Buongiorno, 1984), nurses (Kayton & Freed, 1967), social workers (Sanders et al., 2005) and general practitioners (Davidsen, 2011). To date, however, there have been few attempts to explore the impact on other practitioners delivering psychological therapy within primary care. Despite the rapid expansion of the IAPT programme in England—currently there are estimated to be over 10,500 practitioners and more than 1.6 million people access the services each year—to our knowledge, no studies have investigated the impact on IAPT practitioners of losing a patient to suicide. Given that the IAPT service model is being implemented in multiple countries worldwide (Baigent et al., 2020; Haarhoff & Williams, 2017; Knapstad et al., 2018; Kobori et al., 2014; Naeem et al., 2017), this question is also of international relevance.

### The current study

We investigated the impact on practitioners within IAPT services of the death of a patient through suicide. Specifically, we aimed to explore both the personal and professional impact, the experience of available support, and what helped or might have helped to prepare the practitioner for such an event.

## METHOD

### Sample selection

We adopted a cross‐sectional qualitative design using purposive sampling and an interpretative phenomenological analysis (IPA) methodology (Smith et al., 2009). We recruited participants from IAPT services across the north of England. Service leads distributed an invitation to participate amongst their practitioners. Similar to other IPA studies (Smith & Osborn, 2007; Taylor et al., 2015), our aim was to recruit 6–8 participants. This is consistent with guidelines for sample sizes in IPA studies, acknowledging the doctrine that ‘less is more’, and resisting the pressure to include higher numbers (Hefferon & Gil‐Rodriguez, 2011). To be eligible for the study, participants must have experienced the death of a patient that they had worked with in their role as practitioner in an IAPT service, where the suicide had occurred either during treatment or within 12 months of discharge. Practitioners were asked not to volunteer if the death was subject to an ongoing investigation or a future Coroner's hearing, or if they were engaged in active support or therapy subsequent to the experience.

### Participants

All seven participants were female. Four were working as PWPs at the time of the suicide and three as CBT therapists. The length of time in their role at the time of the suicide ranged from 1 to 5 years (mean = 3, standard deviation [*SD*] = 1.2). The length of time since the death ranged between 2 and 8 years (mean = 5 years, *SD* = 2.0). For all seven participants this was the first and only experience of patient suicide in their current role, although two participants had worked in multi‐disciplinary teams and had been previously exposed to patient suicide in these settings. Three practitioners were required to attend a Coroner's inquest, and all were required to produce formal reports either for the Coroner or as part of their host organisation's serious‐incident investigation procedure. For three practitioners, the suicide occurred during treatment, for a further three it was following discharge, and for the remaining practitioner, the death was after referral to secondary mental health services.

### Procedure

The semi‐structured telephone interviews were planned to last approximately one hour and were audio recorded and then transcribed verbatim. Interviewees received a participant information sheet, a consent form and a copy of the proposed interview schedule (see Appendix A). Participants were advised that the interview schedule was only a guide and was not intended to be prescriptive. The study was approved by the NHS Health Research Authority (IRAS ID 249864) and ethical approval was granted by a University ethics committee in the UK.

### Analysis

We analysed the transcriptions using an IPA approach. IPA is strongly rooted in the lived experience of the participants and strives to understand how an individual makes sense of major life experiences (Smith, 2018). It explicitly acknowledges the ‘double hermeneutic’ of the researcher offering an interpretation of the participant's own account of the significant event, which is the subject of study (Smith, 2011). This explicit acknowledgement was important as the researcher was immersed in a similar work environment to the participants and had previous research experience in the area of study.

The first author (DS) carried out the initial analysis, and it followed six stages: (1) multiple reading of the transcripts; (2) initial noting and attention to the semantic content; (3) development of emergent themes; (4) searching for connections across emergent themes to create an initial list of themes; (5) moving to the next transcript and repeating the first four stages; and (6) searching for patterns across all transcripts, the identification of recurrence of themes across multiple participants and finally the grouping of themes into superordinate themes (Smith, 2011).

To improve rigour and coherence, the third author (RT) audited the analysis (Smith et al., 2009) by examining two fully annotated transcripts. This was to check that the interpretation and coding were credible, that the identified themes were supported by the data and to confirm a logical link through transcripts, themes and superordinate themes. On the basis of feedback from the audit and from the peer review process names of superordinate themes were clarified and further examples of the themes were identified with illustrative quotes added to the text. The second (OJK) and fourth (ROC) authors then agreed the logic of the analysis through discussion with DS following review of the tables of quotes (Table 2).

### Reflexivity

The first author (DS) conducted the interviews. He is a cognitive behavioural therapist (CBT) who has worked in an IAPT service for 10 years and is concurrently a part‐time PhD student. He has previously carried out a systematic review of the impact on mental health practitioners of the loss of a patient by suicide. He has not experienced the loss of patient, friend or relative by suicide, although he has witnessed the impact of such deaths on fellow practitioners.

### Complementarity between IPA and CBT

An IPA approach aims to go beyond the descriptive. Further to the identification of descriptive themes it should develop a deeper interpretative analysis. This is the double hermeneutic mentioned previously. For the current study the description of the participants' personal and professional reactions was important in order that these could be compared to the reactions evidenced in studies of the impact on other mental health practitioners. The IPA approach, however, was employed to gain a better understanding of these reactions with the hope that this could guide improvements in the preparation and support made available to practitioners. DS' CBT background is perhaps relevant here. Core to CBT is the principle that it is not events alone, but rather the idiographic meaning placed on the events, that explain emotional and behavioural reactions. An individual's pre‐existing beliefs, based on their experiential learning, are theorised as offering an explanation for the derivation of meanings of events. Formulating emotional difficulties from a CBT perspective involves understanding how and possibly why an individual interprets events as they do. This mirrors the hermeneutic efforts of IPA, with the researcher presenting an interpretation of the participant's expressed understanding of their experience.

## RESULTS

Analysis of the individual interview transcripts identified a number of emergent themes. Related emergent themes were subsumed (for example ‘perfectionism’ was subsumed into ‘recognition of personal traits’) to establish a list of twelve themes. The identified themes were found to be represented in the majority of the transcripts (see Table 1).

**TABLE 1:** Ubiquity of themes across participants

Theme	Participant							
	1 ‘Amy’	2 ‘Beth’	3 ‘Cath’	4 ‘Donna’	5 ‘Erica’	6 ‘Fran’	7 ‘Gina’	Present in majority
Fear of Blame	Yes	Yes	Yes	Yes	Yes	Yes	No	Yes
Sense of shock	No	No	Yes	Yes	Yes	Yes	Yes	Yes
Upset	No	Yes	Yes	Yes	Yes	Yes	Yes	Yes
Sense of guilt	No	Yes	Yes	Yes	No	No	Yes	Yes
Self‐questioning—doubt	No	Yes	Yes	Yes	Yes	Yes	Yes	Yes
Impact on practice	No	Yes	Yes	Yes	Yes	Yes	Yes	Yes
Search for understanding	Yes	Yes	Yes	Yes	Yes	Yes	Yes	Yes
Recognition of personal trait	Yes	Yes	Yes	Yes	No	No	No	Yes
Benefit of official enquiry	Yes	No	Yes	Yes	No	No	Yes	Yes
Growth and learning	Yes	Yes	Yes	Yes	No	Yes	Yes	Yes
Identification of systemic problems	Yes	Yes	Yes	Yes	Yes	Yes	Yes	Yes
Preparation	Yes	Yes	No	Yes	Yes	Yes	Yes	Yes
Support	Yes	Yes	Yes	Yes	Yes	Yes	Yes	Yes

**TABLE 2:** Themes

Superordinate themes	Incorporated themes	Sample quotations related to theme
Feeling shocked and upset at the death of a patient	Sense of shock	P3 There's the shock and the upset… P4 I was in shock and then I just started crying. Like with any traumatic event that you experience it feels like it was only yesterday. I can remember exactly the day I found out. P5 It was a real shock I found it really upsetting, more than I expected to be upset by… I remember feeling really kind of shaky, I felt physically sick. P6 …and it was such a shock that it happened, when I was delivering guided self‐help with somebody P7 To say that I was shocked, is an understatement

Upset	P3 There is the upset and sadness as well P4 …and it was him who could see how upset I was and said to me, take some time off. P5 I just…yeah, I found it really really upsetting, more than I expected to be upset by it. P6 I remember getting upset about it

Sense of guilt	P7 I was just in shock and a guilt, I suppose. I did feel guilt. P3 I remember knowing that that worry that I had done something wrong was a natural response, but I still really worried about it. Worry that this is my fault and I've done something wrong. P2 …dread feeling that you have done something wrong. I'm in trouble for something. P4, It made me feel like it was my fault. P3 I was the only person who had contact with him. That, you know, if there were a couple of people involved in it, whether that would feel different. P5 …but the big difference, we were all a team, we managed the case load as a team, so I did not have that level of responsibility as such of being the only person that's the point of contact… …this was my case, and he was my patient, and it was just me and I thought this is…I'm responsible for this young lad I did feel a strong sense of responsibility for this young man

Fear/anxiety of potential blame	P2, It felt like there was a black marker against my name, that this suicide has happened, you are blacklisted, kind of feeling to it. Immediately after the event I think I went through phases of being hypervigilant to it … to the avoidance side of it … I was so burnt out I could not kind of deal with it and if I do not ask I do not know and I do not have to do anything about it I suppose. P1 …some doubts creeping in because it went quiet…are people wondering certain things aren't right? It's actually having a lot of trust that your management will do the right thing. That they are not…they are not out to blame or to, yes, pick apart things. P3 This is all on me. Worried that I would be judged and criticised for the decisions that I made. P4 …did make me feel like I was responsible, even though I know I wasn't you do feel like you are on trial. P5 Oh my God, it's going to be awful, they are going to hold me responsible and rip me to pieces. P6 They are looking to blame. That's how it felt, they want to blame someone or a fault I was standing up there for the service. P7 Fear that my practice would be questioned
Attempting to understand the causes of the suicide	Self‐questioning and doubt	P2: What did I miss? Why did not I know that that was going to happen? Why did not I sense, I suppose, that that's how he was feeling and that's what he was thinking? …you know, toothcomb all your notes What's the point anyway, because he scored zeros, he told me there was no risk and yet that happened… P6: …it all came back then, is there something more I could have done? Just lots of questions. P3 …is that on the surface? Was there stuff there? Should I have done something better? Maybe this is my fault? P4 Running everything through, you know, what happened, what did I do, what could I have done differently? And thinking, was it my fault, did I let him down, did I let him down, did I let his family down? P5 …and just panicking that you have done everything right …is it something I've done or something I've not done? P7 I thought, gosh, have I…is it me? Have I done…? You know, maybe if he had not come in for therapy, if we had not discussed…you know the way you do question, sort of, just questioning your practice but…

Search for understanding	P1: …so my reaction over the next few days and week, I guess, I …it was obviously kind of, like sense making in my head, I suppose. P1 If they are going to do this, they are not going to tell you like and you cannot, you are not psychic P2 … generally, more accepting of just the yeah, there's only so much that I can do, and I'm okay…. more okay with that. P3 It is unfortunately something that does happen. We all do everything we can to try to help people, but it does happen, and I guess, that really, has really stuck in my mind I mean people have free will. As good as risk assessments and support that we give, some people are going to end their lives and that is …and I guess that, telling us that we can only do what we can do. P4 We can never know all the circumstances, we can never prevent it from it completely happening. I know that I could not have stopped him doing it. P5 …too good to be true that it's not happened since, you know what I mean? It's like you think the volume of people that we work with and the amount of telephone assessments that we do, and you just… It sometimes can feel quite overwhelming, that, that you think that it's a vast amount of people that you are coming into contact with, and the odds are that it's going to happen again and it's just a matter of when not if. P6 I do feel that at times people make a decision and nothing is going to stop them… but I do feel there are a handful of people where you could change that. P7 …oh my God, what's the reason? There was a reason why, and the reason was..

Impact of official enquiry	P1 I was just absolutely up for having conversations with people about what had happened, where necessary……with somebody where they were doing a bit of an investigation. P3 When I've looked through all the notes I know that I had and I am confident in that decision‐making, my supervisors are. I was absolutely dreading having to go, but there was an element that, kind of, I do not know if closure is the right word, but just to….help me process it P4 (the serious incident report) made me feel like I was responsible for his death, even though I know I wasn't. But when it came out, when we did do the coroner's court, there was no way I could have done anything, I could not have prevented that man doing what he did. ….the serious‐incident report, and she'd come to talk to me about it – me and X – and I think the whole way she talked, or the emails, I think she sent me a couple of emails, and the way she spoke to me kind of upset me. P7 The whole process of having to do a report for the coroner was very painful as well. As hard as it was, yes. I remember thinking, you know what, I actually….I did the best I could

Identification of systemic problems	P4 What inevitably happens is that some of the people who might have been slightly more risky than you'd like might have been better suited in a different team. You felt there was pressure because people could not be accommodated in those other mental health services. …all of us as therapists are pressured to see more and more people P6 …have such a high case load, you see so many people … I soon learned it wasn't what they said it was at Uni and it was more moderate to severe rather than mild P5 I was told that really wasn't the way I should've been informed – someone should've spoken to me properly P4 You know let us not have people jumping down your neck straightaway about reports and the blame culture P7 Why was it right that I've had an email? Why did not …? You know when you can work through a process, why did not somebody have the decency to ring me and say? There did not seem to be any transparency, it was that well, this has happened, and it's only happened to you, whereas obviously this has happened to other people…..
Learning from the tragic event	Growth through adverse event	P2 I learnt lot about myself as a result of that. …less pressure on myself, I'm a lot more compassionate to myself. I guess more accepting around the nature of things rather than taking 100% of the responsibility I definitely have become more confident in managing risk. P4 …but kind of very clear, even quite soon afterwards, a drive to try and learn from the experience, and crucially to try and help others around you. P6 So I feel that I've learnt a lot from that and I have supported people who have had to go to court themselves. P1 …it just gives me more confidence to, kind of, say this…can this happen please? it's like, well, I do not know, with experience….I do not know, would I dig a little bit more, but…I do not know. I'm not sure.
	Impact on practice	P5 you do feel very tentative about discharging people… …and just being a bit more risk averse than you might normally be. I've always been very thorough in my notes and everything and all my risk assessments have always been, you know quite thorough, but perhaps just tightening it up more. perhaps a bit more direct about the way you ask questions to people and talk about it. Overcautious P6 I am very thorough in risk assessments and when it comes to notes. I do share the experience with clinicians P7…it's difficult to positively risk‐take when you have got that in your background. …if anybody had a little bit of risk, I'd really jump on it. But I think I've definitely got that balance back. Yes, so your history of contact with clients definitely does change how you approach risk with other clients
Reflections on what helped in coping with the tragic event	Recognition of personal traits	P2 …that's all kind of bringing out obviously my own negative kind of beliefs about myself that does not fit a perfectionist's kind of world. P3 I always have that with everything in life, just generally there in the background that if something goes wrong then it's probably my fault. P4 I'm the kind of person who tends to try and push through things……it probably took someone else to tell me to take time off

Preparation	P1 If you expect that this is never going to happen you are living with a false reality aren't you because it will, it will P2 Bizarrely felt quite naive to the actual possibility of that happening. P4 But it is one of those things I think everybody who works in the field, mental health, dreads. So why not address it before, rather than wait for it to happen, or worry about it happening, why not prepare people a bit more. P5 …just to maybe have people more prepared for the impact of what, you know, possibly…what to expect about, you know, and just perhaps somebody that's had the experience just to talk a little bit about it. P6 …if there were more training and people came in and talked of their own experiences, people could use that knowledge and reflect and make them more aware in sessions maybe. P7 I just think there is such an importance on making people aware a, that it can happen and what to expect if you go to court because that's quite scary. P7 I think we could do probably do with a little more risk training as well. I think that might be helpful. P1…really direct stuff about this in training

Helpful support	P5 …I suppose, the supervisory relationship has been very helpful with that [sense of responsibility].‘and I remember P2 …talking about it quite a lot in supervision as well… P3 Everybody is incredibly open about everything that we go through and everyone kind of leans on each other and we do not feel there is any hierarchy amongst colleagues and therapists. So, it is a very supportive team in that respect. P6 …just checking in, not just, oh you have written a report let us forget about it. P1 There was a couple of days at least, I think, two or three days where it went a bit quiet… and I remember there just little … some little doubts creeping in because it went quiet. P6 …some training around writing a report would have been really helpful. P1 We should, probably, be asking how each individual would want…[to be kept informed] P4 A procedure written down for people to follow and say, first of all, does that person need to take time off, or what does that person need? P5 having something like that as an actual procedure where it would just happen without you having to ask. *Importance of validation of response:* P3 Yeah, or appreciate that this might be a bit upsetting for me to do. P5 Well it was just nice to have somebody to kind of just validate what I was feeling about it really, that was the main thing. P7 It's like having bad news in a hospital, you just need to sit with the person and validate their experience and I suppose be empathetic and all that kind of compassion focussed stuff and as you know

Following this, we grouped the themes together to create four superordinate themes: (1) feeling shocked and upset about the death of a patient; (2) attempting to understand the causes of the suicide; (3) learning from the tragic event; and (4) reflections on what helped in coping with the tragic event. The themes were incorporated into each of the four superordinate themes and representative quotes are shown in Table 2.

### Feeling shocked and upset about the death of the patient

The four main emotional reactions described by the participants were shock, upset, fear of blame and guilt.

#### Sense of shock

Five practitioners described a sense of shock when they were informed of the death. For example, Donna:

I was in shock and then I just started crying…

and Fran:

…and it was such a shock that it happened, when I was delivering guided self‐help with somebody.

Notice the emphasis here on the treatment type ‘guided self‐help’ (an intervention for relatively mild to moderate presentations of anxiety or depression). This may have been to communicate the practitioner's expectation that risk would be proportionately low in their patient group. We elaborate further on this in the examples we provide later in the subsection describing preparation and support.

In contrast, one practitioner, Amy described managing the initial impact:

‘I remember feeling lots of things in one go, but, also, how can I say it? It wasn't overwhelming…’ …. ‘I remember it was…feeling quite a big reaction, but not that it was overwhelming really’.

Amy acknowledges the extent of her initial reaction but is also careful to repeat an expression of her ability to cope. This ability to not feel overwhelmed is interesting and was despite this practitioner also stressing the unpredictability of the suicide:

‘….there was no clear indicator. There was no indicator that… that's what had happened really or was going to happen.’ …. ‘…and had been consistently better and he was scoring zero to risk questions…’.

Amy communicates clearly that the problem was an absence of warning signs rather than a lack of diligence on her part and this understanding would appear important in coping adaptively. Amy went on to reflect on her own tendency to act quickly:

‘…my nature to be a bit, kind of, like, okay, action stations, this is something…yes, this is something to, kind of… this is something to face and deal with…’ ‘…wow this is a new thing. This has never happened before. This is something new to try and manage.’

It could also be due to confidence in having followed the service risk protocol:

…but what helped me to be fine with it was that I knew I had done the best I could and the right things that I needed to do at the time…

I think because I knew I had done what I had supposed to do that made it possible for me to manage the situation.

Amy's account appears to communicate that she has a clear understanding of her role and responsibilities. For her this entails knowledge of the correct protocols combined with a professionalism that strives to fulfil that role but also copes with and learns from adversity. We might speculate that this was protective against the self‐questioning, doubt and fear of blame experienced by other practitioners. However, we will describe later how this was undermined for Amy as a consequence of lack of communication.

#### Upset

All the practitioners apart from Amy described being upset. For most this was described as part of the initial reaction, as with Gina:

It was both shocking and upsetting in equal measure…

whilst for others this upset was reported later as a consequence of the enquiry.

In comparison, it is notable that Erica described her feelings of shock and upset being greater than she had expected.

I found it really upsetting, more than I expected to be upset

This indicates prior contemplation of the possibility of losing a patient to suicide and, therefore, an awareness of this risk. This is understandable in this context as Erica had previous experience in a multi‐disciplinary secondary care team who worked with individuals presenting with high levels of suicide risk.

#### Fear of blame

Fear of blame was cited by all of the participants apart from Gina, although there were variations in the ascribed source of this. For some, this appears to have been predominantly self‐generated, related to a reflective process or characteristic trait as described by Cath:

‘This is all on me.’…‘Worried that I would be judged and criticised for the decisions that I made.’

I always have that with everything in life, just generally there in the background that if something goes wrong then it's probably my fault.

Notice that whilst Cath had the awareness of her own propensity to self‐blame, this insight did not protect her from the presumption of being at fault and fearing that she would be held to account.

For others the source of the fear of blame was seen as more external and as a consequence of the work environment or processes of investigation. One practitioner (Amy) found that lack of communication from her managers during the time that the death was being investigated had contributed to rising fear of blame:

…some little doubts creeping in because it went a bit quiet…

…are people wondering certain things that aren't right, that I'm not aware of?

and this was despite her early and ongoing confidence that she behaved correctly and professionally as noted earlier. Amy's account can also be viewed as highlighting the difference between feelings of guilt, i.e. linked to an interpretation that an event was caused by personal acts of commission or omission and of a fear of blame i.e. that the cause will be attributed to you despite your actions. Both the ubiquity of fear of blame and the way that it was expressed could be interpreted as indicators of what is often perceived as a ‘blame culture’ within organisations.

This can be heard from Beth for whom the response from the service had also increased the fear,

It felt like there was a black marker against my name, that this suicide has happened, you're blacklisted, kind of feeling to it.

Oh my God, it's going to be awful, they're going to hold me responsible and rip me to pieces

Both these examples indicate the uncertainty felt in the time following the death and how clinicians attributed this to lack of communication or insufficient support. Gina, the only participant not to describe fear of blame, said that her former experience in leadership had equipped her with knowledge of the correct procedures that should be followed subsequent to a death. When Gina perceived that these had not been followed, her initial shock and upset changed to feelings of anger and of having been let down, particularly in how she had been informed of the death:

I actually put an email into the service lead about the process and said that I found it really distressing and hopefully people could learn.

In some cases, however, the fear of blame was compounded by examples of actually feeling blamed e.g. through the internal investigation or as a consequence of the management of the Coroner's hearing, despite the participants referencing evidence of the injustice of this.

They are looking to blame. That's how it felt, they want to blame someone or a fault….. I was standing up there for the service. (Fran)

‘Like with any traumatic event that you experience it feels like it was only yesterday. I can remember exactly the day I found out.’ …. ‘it did make me feel like I was responsible, even though I know I wasn't, you do feel like you're on trial.’ (Donna)

These accounts suggest that practitioners' felt confident in their professional competence. However, feelings of doubt and fear of being blamed could still be raised as a consequence of their experiences subsequent to the death.

#### Sense of guilt

The majority of participants in this study described feelings of guilt. These could be categorised in three areas; (i) concerns over potential acts of omission, (ii) acts of commission:

is it something I've done or something I've not done? (Erica)

Or (iii) as with Amy, thoughts related to their emotional reaction:

it was all about them… and it's me, kind of, talking about my stuff.

Two practitioners described how the nature of their role as sole practitioner led to a heightened sense of responsibility contributing to feelings of guilt.

I was the only person who had contact with him that you know, if there were a couple of people involved in it whether that would feel different. (Cath)

Erica explained the change in sense of responsibility from her former role within a different mental health service:

we were all a team…we managed the caseload as a team…so I didn't have that level of responsibility as such of being the only person that's the point of contact for patients.

Given the rarity of suicide and the caring role that the practitioners fulfil it is understandable that they would experience shock and upset. However, listening to their accounts makes it clear that the impact was deeper and more prolonged than a natural empathetic response.

### Attempting to understand the causes of the suicide

This superordinate theme represents a process that all practitioners described in their attempts to resolve their reaction to this novel and distressing experience. Themes within this were self‐questioning and doubt, searching for understanding, identification of systemic service problems and the impact of the formal enquiry.

#### Self‐questioning and doubt

Self‐questioning and doubt could be viewed as related to the previous theme of guilt and self‐blame:

…so I was really blaming myself, I suppose, for missing something beforehand. (Beth)

However, the examples included within this theme represented the need to understand what had been a shocking and, therefore, unexpected event:

That confused me more….so what did I miss, what ….yeah, how did I not know that it was as bad as that? (Beth).

Notice here the dilemma expressed by Beth and mirrored by other practitioners. Either they had not followed risk assessment protocols effectively or the risk assessments themselves were fallible. Cath explained how she had resolved this:

As good as risk assessments and support that we give, some people are going to end their lives and that is …and I guess that, telling us that we can only do what we can do.

#### Search for understanding

Practitioners described further attempts to both understand the cause of the suicide and how the level of risk had not been detected through assessment and treatment. Descriptions were offered of attempts to understand what had happened from the perspective of the person who had died, although the specific circumstances differed. One practitioner, Gina, for whom the person had been discharged many months previously, described the need to understand the factors that had contributed to the suicide and whether these related to therapy:

I just felt very, very sad for him. I felt very, very sad and also relieved that it was nothing….well as far as I know, it was nothing to do with the therapy that I'd done with him.

For Gina, the access to further information relating to the circumstances of the death had enabled her to resolve fear of being at fault or in any way responsible.

For Erica, the suicide had occurred after the individual's care had been transferred to secondary services due to suicidal behaviour. Therefore, the level of risk had already been identified and understood:

I wasn't actually surprised that he did go on to complete suicide after a period of time.

Although Erica's former role had exposed her to the death of patients by suicide, that experience had been in a team context rather than as the sole practitioner in contact with the person who died.

…in that team you used to just be relieved if you weren't the last person, and I know it sounds awful but as long as you weren't the last one to have seen the person, you used to have this sense of relief….

and given that the patient had been recently discharged:

And it was that kind of feeling that I knew he'd been seen by one of the mental health liaison nurses prior to the end…after I finished my input with him, but it's still….yeah, it was really upsetting.

Here again, arriving at the understanding that she was not responsible had brought relief from fear of blame and enabled her to focus on the sense of loss and upset.

Five practitioners commented more generally regarding their beliefs about the efficacy of suicide prevention efforts, and although it was not always clear if these indicated a change in attitude following the experience of loss, there were examples of these helping the practitioners resolve what had happened.

We can never know all the circumstances, we can never prevent it from it completely happening. …

I know that I could not have stopped him doing it. (Donna)

#### Identification of systemic problems

Respondents highlighted problems with the service or the wider organisation as compounding factors. It is possible that identifying these wider factors helped to mitigate the sense of guilt and fear of blame. These included the level of complexity of the presenting problems of the people they worked with, the associated risk, the lack of more appropriate services and the volume of workload.

… I soon learned it wasn't what they said it was at Uni and it was more moderate to severe rather than mild (Fran)

A clear indication from Fran of her expectations to be working with people experiencing less complex mental health problems and, by association, lower levels of risk.

#### Impact of the official enquiry

Five participants described the formal review process as helpful in clarifying that they had carried out their duties correctly and in processing the event. Cath, for instance, explained:

When I've looked through all the notes I know that I had and I am confident in that decision‐making, my supervisors are…

I was absolutely dreading having to go, but there was an element that, kind of, I do not know if closure is the right word, but just to….help me process it

However, there were notable exceptions to the benefits of a formal review as three participants described how the investigations had significant negative impacts:

It kind of felt a little bit like being …kind of put… being put at the stocks in a sense….

…You're taken to court and you are kind of punished for it.

…and so it very much didn't feel like it was what it was supposed to be in terms of actually finding out the facts of what happened and don't feel like really we got any further clarity (Beth).

All practitioners described their attempts to understand the loss of life through suicide. This helped to find some resolution of the emotional impact; however, this was either helped or hindered by formal reviews.

### Learning from the tragic event

#### Growth through adversity

This theme incorporates the theme of growth through an adverse event:

…but kind of very clear, even quite soon afterwards, a drive to try and learn from the experience, and crucially to try and help others around you (Donna).

This determination to use the experience to support others was mirrored by Fran, whilst Beth described a sense of personal growth:

I learnt lot about myself as a result of that.

#### Impact on practice

Practitioners described some positive influence on risk management:

I am very thorough in risk assessments and when it comes to notes. (Erica)

…and as the years have gone on, I'm not so anxious, I am just more aware. (Fran)

Two practitioners mentioned that they observed learning and improvements at the service level although it was not clear whether these changes had been a consequence of their experience or not:

‘…but I don't feel like it was as clear necessarily as it would be today and I imagine that it would be handled a little bit differently…’ ‘…but I wonder if now, just again through the experience over the years, I suspect, that maybe now it would be this is what happens…’

….systems, protocols, procedures have developed. (Amy)

Examples of impact on practice were in some cases short term:

Just distraction. Because I was so focussed on making sure I got a thorough risk assessment, I perhaps wasn't doing as much therapeutic work. (Fran)

Immediately after the event I think I went through phases of being hyper‐vigilant to it … to the avoidance side of it … I was so burnt out I couldn't kind of deal with it and if I don't ask I don't know and I don't have to do anything about it I suppose. (Beth)

This also indicates a loss in confidence in dealing with risk and in the process of risk assessment itself:

What's the point anyway? He told me no risk and yet that happened

For Beth, however, this was also temporary:

I definitely have become more confident in managing risk.

In summary, although some practitioners described negative changes in their management of risk, these were temporary, and all reported deriving learning from the tragic event.

### Participants' reflections on what helped in coping with the tragic event

This encompasses three themes: recognition of personal traits, preparedness and helpful support.

#### Recognition of personal traits

Examples of personal traits that were seen as contributing negatively to the impact were perfectionism, pre‐existing negative self‐beliefs, a tendency to self‐blame and an attitude of fortitude that interfered with self‐care and help seeking (see Table 2).

#### Preparation

Participants offered suggestions that might have helped them be better prepared for a patient suicide, some of which they were able to put into practice to help others in the future. These included increasing awareness of the potential for patient suicide, and more openness and communication about serious incidents,

Just knowing that it does happen… because I always felt like I was the only one because it wasn't really talked about (Fran)

Practitioners also cited the importance of being confident in following the correct procedure for risk assessment and management.

#### Support

All practitioners described benefiting from the informal support of their direct team and colleagues:

…and getting loads of support, a lot of things, from other members of the team and stuff like that.(Erica)

I would say it has always been a pretty cohesive and supportive team. Well, actually I will take the pretty out of that, a really cohesive supportive team (Amy)

although two described contact with managers as less helpful:

I guess we were struggling to adjust to a new manager with a different style.

the high people and high managers that you never see but you get those emails from… (Cath)

Three practitioners mentioned the benefit of having their reactions to the experience validated; they felt supported. The importance of the supervisory relationship was also mentioned.

I suppose, the supervisory relationship has been very helpful… (Erica)

However, note the ambivalence in Erica's comment. This and the fact that formal supervision was not cited as a source of support by other practitioners is surprising given the emphasis placed on regular supervision within IAPT services.

Having their training needs met was identified in three cases as important (e.g., in report writing and risk training).

Yes, sometimes I think we don't do enough, really, about risk. I know it can be very inexact trying to manage risk but I think we could probably do with a little bit more risk training as well. I think that might be helpful. (Gina)

Notice also here that Gina refers to the potential fallibility of risk assessment and management protocols.

Clarity about support procedures was also suggested by Donna and Erica, although one practitioner (Amy) indicated that an individual approach to identifying support needs was desirable.

The descriptions related to this theme highlighted the diversity of practitioners' experience but also the individualised nature of support needs. The benefit of raising awareness to the possibility of loss of a patient through suicide to better prepare practitioners was, however, reflected on by all but one participant (Table 2).

## DISCUSSION

Our study explored the personal and professional impact on practitioners within IAPT services following the death of a patient through suicide and practitioners' experiences of preparation and support. The practitioners described the initial emotional impact, a period of resolving the experience, longer‐term consequential changes and aspects that they found mitigated the impact. A previous qualitative review of the impact of patient suicides on psychiatrists, nurses, social workers and mental health workers on an inpatient unit identified three phases of experience reported by staff (Cotton et al., 1983). Evidence for each of the phases could also be identified in the accounts by the participants in the current study.

These phases were (1) working in shock (2) emergence of overwhelming feelings and (3) new growth over emotional scars. The main initial emotional impacts described in the present study were upset, shock, guilt and fear of blame. Practitioners reported becoming more cautious in managing risk. Previous studies involving other mental health professionals have reported comparable findings. The main reactions of nurses have been described as shock, fear, guilt and stress (Wang et al., 2016). Social workers reported sadness, shock, self‐blame, anger and fear (Sanders et al., 2005) whilst psychologists indicated shock, anger, guilt, distress and sadness (Finlayson & Graetz Simmonds, 2016). One study (Séguin et al., 2014) reported, in contrast to others studies, that although practitioners did report shock, helplessness and sadness the overall emotional impact and reaction was low. The authors of that study suggested that a number of features of their sample might explain this finding and indicate protective factors. These included that the practitioners reported receiving sufficient support, they worked in a team setting and had experienced a number of suicides with a potential habituation effect. The current study provides some support for this explanation as the impact here was related to problems with support, having sole responsibility and lack of previous exposure.

### Preparing practitioners

Following a traumatic event, the individual has to either assimilate or accommodate the novel experience into their existing understanding (Ehlers & Clark, 2000; Resick et al., 2017). Prior beliefs held by practitioners could interfere with their ability to cope with the experience of losing a patient to suicide and contribute to psychological and behavioural disturbance. Such unhelpful beliefs could include: that their role (and the task of risk assessment) held the sole responsibility to prevent all deaths, that the elimination of all suicide was achievable, that the service they worked for was for people of low risk, therefore, suicide was extremely unlikely, or as a practitioner, they had sufficient efficacy to prevent all suicides on their caseload. If the experiences of losing a patient to suicide were shared amongst the wider team the knowledge that such events occur would help prepare practitioners and other unhelpful beliefs could be shifted (i.e., that ‘competent practitioners do not lose patients’ or ‘I am the only one this has happened to’) and ultimately lead to a cultural shift. Barriers to sharing experience should be explored and addressed, for example belief in this being a taboo subject, fear of shame or blame, collective silence or lack of opportunity. An example of services' response to this problem is the introduction of Schwartz Rounds (Robert et al., 2017), which are designed to give all staff a forum to discuss the emotional impact of working in health care. Likewise a system of peer support to encourage practitioners to seek help, such as in the Trauma Risk Management (TriM) approach, could be adopted (Greenberg et al., 2010).

### Mitigating the sense of guilt

Helping mitigate practitioners' sense of guilt could be informed by Kubany's model for the understanding and treatment of trauma related guilt (Kubany & Manke, 1995; Young et al., 2021). This highlights four potential cognitive biases: hindsight‐bias (i.e., presuming that current knowledge of the outcome was known at the time of the event) ‘Now I know what happened, if I'd had that knowledge when I went back, you would do something different because you know the outcome’; responsibility (i.e., taking on all or most of the responsibility for the event) ‘this was my case and he was my patient and it was just me and I thought this is…I'm responsible for this young lad’; justification (i.e., believing there was no justification for choices taken at the time and ignoring the conditions under which you took those choices) ‘….judged and criticised for the decisions I made’; and wrongdoing (i.e., the belief that you have violated personal values) ‘dread feeling that you've done something wrong’. The underlying themes of each of these categories of bias could potentially be addressed either with the individual practitioner or for example, to identify targets to be incorporated in the educational component of risk management training.

The function of self‐questioning and self‐doubt could be explained by the need to understand what had happened in order to better prevent it happening again (therefore, fulfilling the professional role whilst avoiding a future aversive experience). However, if performed in a maladaptive way, there can be a close link between these processes and the emotional reaction of guilt. This could be viewed as parallel to the potential impact of a formal review; if carried out in the spirit of learning rather than blame attribution, the processes can helpfully contribute to resolution.

### Fear of blame and just culture

Within the NHS and in health and social care organisations more generally the need to shift from a blame culture to a just culture has long been recognised. The shipping and aircraft industries have been seen as exemplars of this approach. Encouraging an openness to discussing problems and mistakes enables learning from adverse events and the promotion of safer systems. The ubiquity of fear of blame amongst the practitioners in the current study would indicate that more needs to be done to move away from the perception of a blame culture. The adoption of a just culture could help reduce the negative impact on practitioners and promote learning and growth.

### Barriers to recovery

A number of factors could combine to hinder a practitioner's ability to cope with and recover from the experience of a patient dying by suicide. An initial problem could be insufficient preparation characterised by a lack of prior knowledge or clarity (i.e. patient's level of suicide risk, extent of responsibility for risk management, required process following a death, likely impact, support available). As highlighted elsewhere, we need to better prepare practitioners for such losses (Bowers et al., 2006; Chemtob et al., 1988; Gibbons et al., 2019; Hendin et al., 2000; Jacobson et al., 2004; Sherba et al., 2018; Wang et al., 2016; Wurst et al., 2011). This lack of preparation could combine with personal characteristics (e.g. tendency to self‐blame, perfectionism, reluctance to seek help) and negative perceptions of the organisational culture (i.e. blame based) may contribute to the reactions of shock, guilt and fear of blame. These reactions will be moderated in turn by factors such as the organisational culture (including degree of openness, support available and the manner in which formal processes are conducted) and the extent to which these contribute in a restorative way to help the practitioner resolve the experience and promote growth and learning.

The finding from this study that all participants valued and benefited from informal and peer support is reported extensively in the existing literature (Pieters et al., 2003; Trimble et al., 2000). Some studies describe this form of support as being the most highly valued (Alexander et al., 2000; Cotton et al., 1983). In contrast, whilst in the current study only one participant discussed the benefit of supervision, previous studies have described individual supervision as being helpful and the most valued of the formal processes in place (Courtenay & Stephens, 2001; Kleespies et al., 1990; Ruskin, 2004; Trimble et al., 2000).

A formal process, if conducted in a collaborative and supportive way, can be helpful to counter doubt, the sense of guilt, fear of blame and to foster a spirit of learning through adverse events whilst guarding against hollow reassurance. Conversely, however, the process can be experienced as fault finding, punishing or shaming if carried out in a less supportive manner. The evidence from previous studies for the benefit of formal processes has indeed been mixed; some finding critical incident debriefs or case reviews useful (Alexander et al., 2000; Kleespies, 1993; Kleespies et al., 1990; Landers et al., 2010; Pieters et al., 2003; Rothes et al., 2013; Sherba et al., 2018), whilst others reported them unhelpful (Bowers et al., 2006; Courtenay & Stephens, 2001, Hendin et al., 2000) and insensitive or persecutory (Gibbons et al., 2019).

On balance, formal reviews are likely to be beneficial if they promote understanding and learning and, therefore, help overcome the sense of shock and self‐blame. However, clearly the manner and spirit in which the review is performed is crucial, so as not to compound or create feelings of guilt or fear of blame.

### Limitations

A potential limitation of the study arises from the sampling process. It is possible that those who had been most distressed may have been unwilling or unable to come forward. All participants were female, however this reflects in part the predominantly female IAPT workforce (NHS England and Health Education England, 2016). Furthermore, there is no conclusive evidence to suggest variation in practitioners' responses based on gender (Sandford et al., 2020).

Integral to the IPA approach is the double hermeneutic; the researcher is interpreting and giving meaning to the interpretation of the event by the participant. As such, it is important to acknowledge how the researcher's experiences will influence this interpretation. On reflection two points seem particularly relevant, firstly the researcher has worked in an IAPT service as a CBT therapist and has first‐hand experience of the impact on colleagues of the loss of a patient through suicide. As a therapist, you are aware that your role is to try to understand any relevant maladaptive meanings that people attach to events and to help the person reappraise these to reduce the emotional impact. In the role of the interviewer, the researcher was acutely aware that the participants had been through the process of resolving the experience, and the goal was to understand this rather than effect change. One possible outcome was that the researcher was perhaps reluctant to pursue particular lines of questioning for concern of destabilising adaptive interpretations. This may have impacted on the richness of the data gathered. Additionally, the interviewer had previously carried out a systematic review on the impact of patient suicide on practitioners. This raises the possibility that the interview questioning was primed to look for evidence that supported previous learning at the expense of a fully objective approach.

### Recommendations

There are a number of recommendations arising from this study. Practitioners need to be clear on the extent of their responsibility and supported to develop the confidence to actively engage with the patient in meaningful discussion around risk. If practitioners are confident in the process of risk assessment formulation and safety planning that guidelines recommend (Department of Health, 2007), they are more likely to engage in it effectively and less likely to be adversely affected in the event of a suicide. Key recommendations that could help mitigate the impact of patient death on practitioners include provision of training that increases awareness of the potential impact and support available in the event of the loss of a patient by suicide and that services develop cultures that support learning after adverse events. For a full list of recommendations, see Table 3.

**TABLE 3:** Recommendations

Delivery	Recommendations
Risk training by the providers of the initial core professional training for this group of staff should cover:	The recognition of the likelihood of experiencing a patient suicide. (i.e. the ‘career prevalence’ of such an event). The limitations of risk assessment tools. The dynamic nature of risk (e.g. risk maybe low when a person is allocated to PWP but circumstances could quickly change). The severity and complexity of presentations within services such as IAPT. (Still widely understood to be categorised as ‘mild to moderate’)
In service training should include:	Clear understanding of the service and statutory procedures following a suicide. Training in risk assessment, formulation and safety planning, including recognition of the poor predictive power of risk assessment tools and the importance of the therapeutic encounter to enable open discussion of risk. Clear guidance on self‐care and support available in the event of a patient death
Services should develop:	An open learning culture. A recognition of shared responsibility. A clear procedure for communicating to the practitioner (by those with direct management or clinical responsibility for that person) in a caring and supportive manner. The opportunity for the practitioner to state their preference for support. Full support for report writing. Ongoing regular feedback during the process. Offer access to a colleague volunteer with personal experience of the loss of a patient

## CONCLUSION

The impact on IAPT practitioners of the death through suicide of a patient appears to be consistent with that found in studies of mental health practitioners more broadly (Sandford et al., 2020). It is hoped that the current study will help raise awareness of this amongst practitioners such as those working in IAPT services and training centres, and encourage them to consider how best to prepare and support practitioners in this eventuality. In the words of Donna:

It is one of those things I think everybody who works in the field, mental health, dreads so why not address it before, rather than wait for it to happen, or worry about it happening, why not prepare people a bit more.

## AUTHOR CONTRIBUTIONS

**Olivia J Kirtley:** Supervision; writing – review and editing. **Richard Thwaites:** Conceptualization; methodology; supervision; validation; writing – review and editing. **Rory C. O'Connor:** Supervision; writing – review and editing. **David Sandford:** Conceptualization; data curation; formal analysis; investigation; methodology; writing – original draft.

## CONFLICT OF INTEREST

The authors have declared no conflict of interest.

## Data Availability

The data that support the findings of this study are available on request from the corresponding author. The data are not publicly available due to privacy or ethical restrictions.

## APPENDIX A

Prior to interview it will be discussed if the practitioner has experienced more than one loss of a patient (the number will be recorded). In this circumstance the practitioner will be asked to consider their response based on the first death through suicide of a patient that they experienced.

Background/context

**Question: Can you describe your work role for me, and broadly speaking the organisation you work in?**

Narrative/Own reaction

**Question: Please can you describe your experience of losing a patient to suicide?**

Prompts:

What was your contact with the person? (e.g. no of sessions, time pefriod)

What was your last contact?

How did you first hear that this person had taken their life?

Can you describe your immediate reaction?

And in the following days/weeks/months?

What was the impact on you personally?

On your behaviour, thoughts, emotions, feelings?

What are the factors that you think most account for the impact you experienced?

Reaction of others

**Question: Can you tell me more about how the response of others influenced your reaction to the death?**

Prompts:

What was the effect on you of their response?

Do you think there has been a longer‐term impact on your co‐workers?

How did your managers respond?

How supported, both formally and informally did you feel and by whom?

**Question: Can you tell me more about the reaction of your family and friends?**

Prompts:

What was the effect of their response on you?

How supported, both formally and informally, did you feel and by whom?

Subsequent change

**Question: Have you noticed any changes in your professional practice since, both in the short term and in the long term?**

Prompts

Has the experience brought about any changes to your approach at work?

In what way has this changed your attitude to suicide prevention?

In what way has this experience changed your confidence when assessing and managing risk?

**Question: Had anything helped prepare you for this experience?**

Prompts:

Prior to this experience had you considered the possibility of losing someone to suicide?

Had any training supervision or advice helped prepare you?

**Question: How do you think people should be supported?**
